# Supplementary material for: Primary Human Trabecular Meshwork Model for Pseudoexfoliation
Source: Cells. 2021 Dec 7;10(12):3448. doi: 10.3390/cells10123448 (PMC8700223; doi:10.3390/cells10123448)
Supplement: Supplementary file 1 [file cells-10-03448-s001.zip › cells-1474962-supplementary.pdf]

SUPPLEMENTARY FIGURES

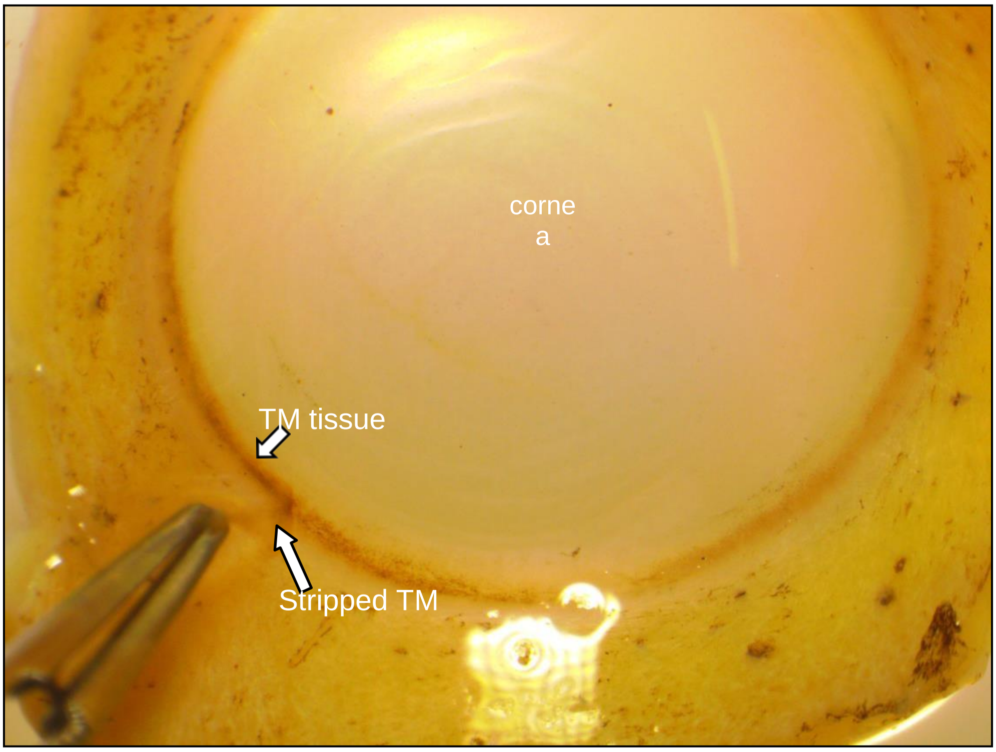

Figure S1: TM stripping from donor corneas under stereomicroscope.

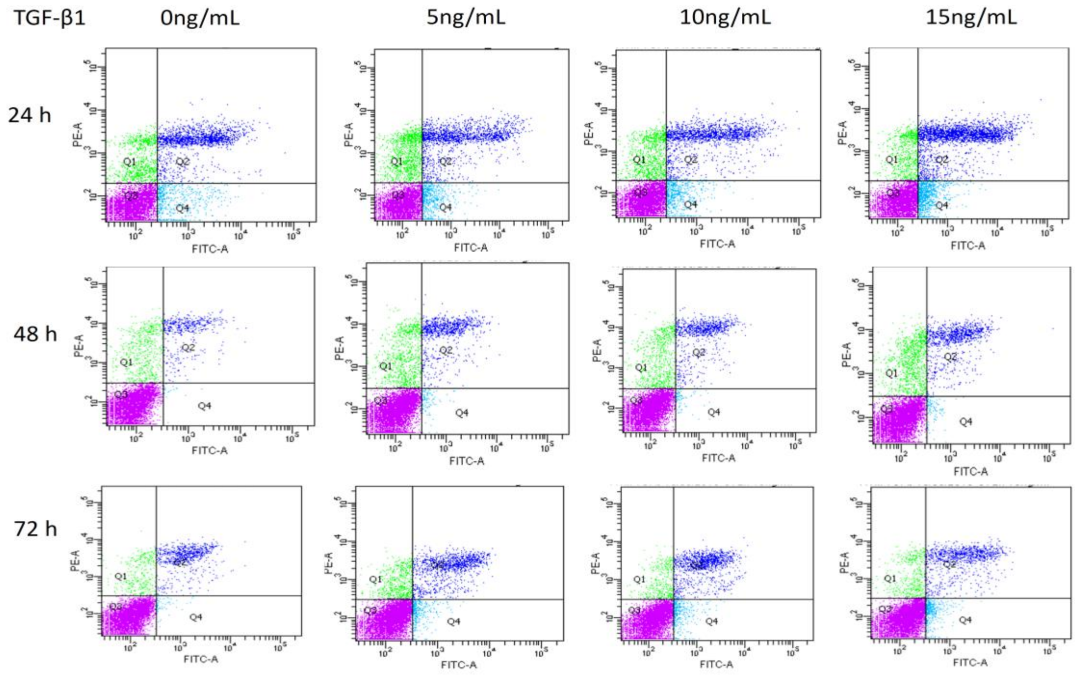

Figure S2: Apoptosis analysis by FACS after TGF-β1(5ng/mL, 10ng/mL, 15ng/mL) treatment for 24 h, 48 h and 72 h.

(a) (b)

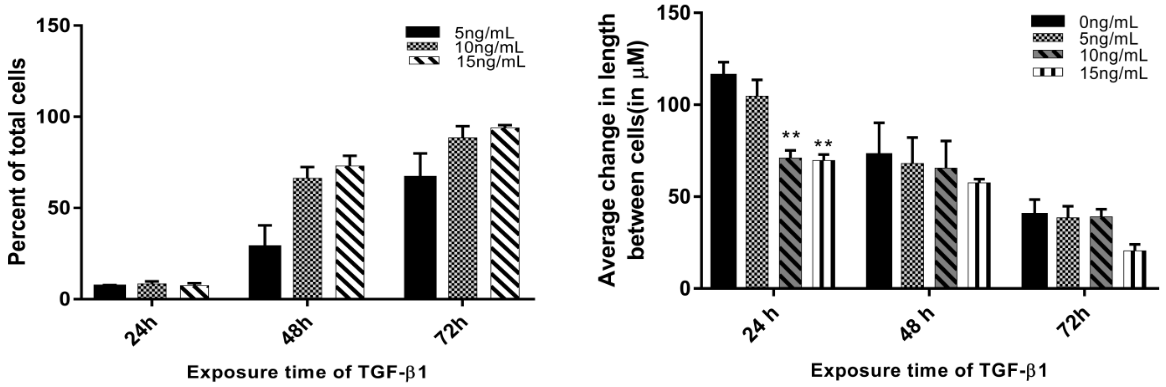

Figure S3: Morphological changes in TM cells after TGF-β1 treatment quantified using ImageJ software(a) percent of total cells undergoing change in length compared to control cells, (b) average change in the length between cells in control and treated group.

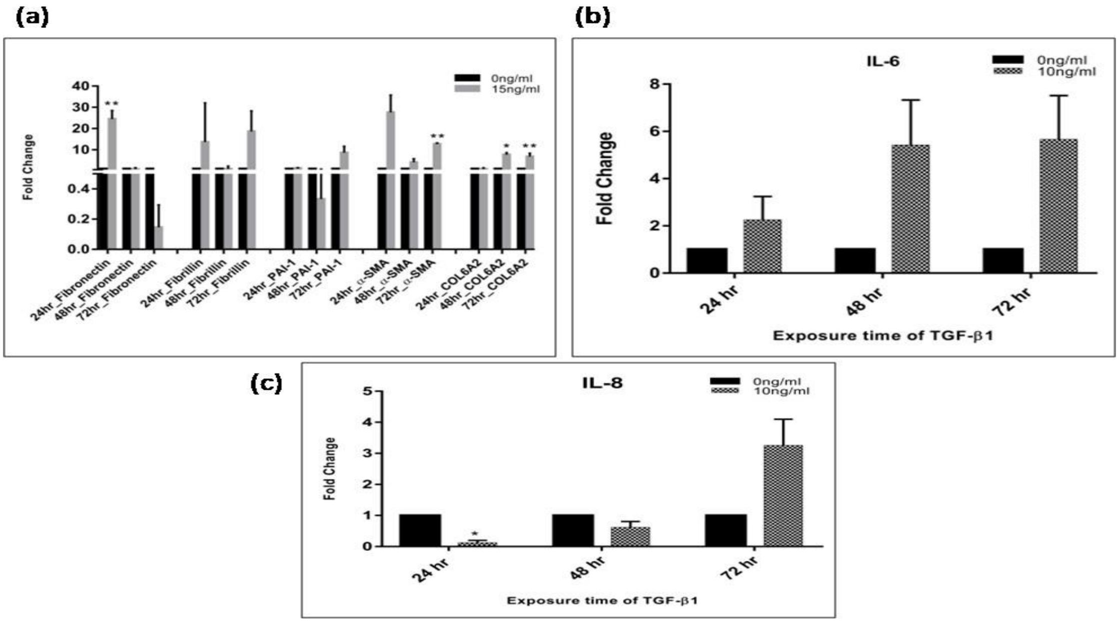

Figure S4: relative mRNA expression of(a) various ECM genes quantified by quantitative RT-PCR after 15ng/mL of TGF-β1 exposure, (b) IL-6 quantified after 10ng/mL of TGF-β1 exposure and (c) Il-8 quantified after 10ng/mL of TGF-β1 exposure

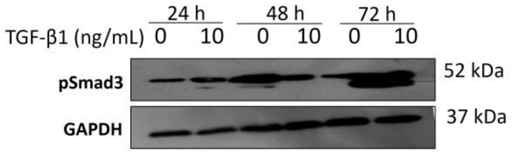

Figure S5: phopho Smad3 levels in control and TGF-β1 treated TM cells.
